# Sinonasal Characteristics in Patients with Obstructive Sleep Apnea Compared to Healthy Controls

**DOI:** 10.1155/2017/1935284

**Published:** 2017-05-04

**Authors:** Mads Henrik Strand Moxness, Vegard Bugten, Wenche Moe Thorstensen, Ståle Nordgård

**Affiliations:** ^1^Department of Otolaryngology, Aleris Hospital and the Norwegian University of Science and Technology, Department of Neuroscience, Trondheim, Norway; ^2^Department of Otolaryngology, Head and Neck Surgery, St. Olavs Hospital and the Norwegian University of Science and Technology, Department of Neuroscience, Trondheim, Norway

## Abstract

*Background*. The difference in nasal obstruction between OSA patients and healthy individuals is not adequately documented. Our aim was to describe the sinonasal quality of life and nasal function in OSA patients and healthy controls using the sinonasal outcome test-20 (SNOT-20), nasal obstruction visual analog scale (NO-VAS), and peak nasal inspiratory flow (PNIF).* Methodology and Principal*. Ninety-three OSA patients and 92 controls were included in a case-control study from 2010 to 2015.* Results*. Mean SNOT-20 score in the OSA group was 1.69 (SD 0.84) compared to 0.55 (SD 0.69) in controls (*p* < 0.001, 95% CI [0.9, 1.4]). The mean NO-VAS score was 41.3 (SD 12.8) and 14.7 (SD 14.4) in the OSA group and controls, respectively, (*p* < 0.001, 95% CI [22.7, 30.6]). PNIF measured 105 litres/minute in the OSA group and 117 litres/minute in controls (*p* < 0.01, 95% CI [−21.8, −3.71]). There was a positive correlation between subjective nasal obstruction and change in PNIF after decongestion in the control group alone.* Conclusions*. OSA patients have a reduced sinonasal QoL and lower peak nasal inspiratory flow compared to controls. Treatment of nasal obstruction in OSA patients should be made a priority along with treatment of the ailment itself.

## 1. Introduction

Sinonasal complaints are associated with obstructive sleep apnea (OSA) [1], and the relief of sinonasal obstruction has been shown to reduce subjective complaints of daytime sleepiness [2]. Excessive daytime sleepiness is one of the main symptoms in obstructive sleep apnea syndrome and a major concern due to the strong association with a reduction in motor skills such as handling vehicles and machines [3]. Even though sinonasal complaints have been described within an OSA cohort, there is still little information on the extent of complaints compared to the normal population. The primary goal of this study was to compare sinonasal quality of life (QoL) in OSA patients with a group of healthy controls. The secondary aim was to compare symptoms and nasal airflow in the two groups.

## 2. Materials and Methods

The study was designed as a prospective case-control trial and was approved by the Norwegian Regional Committee for Medical Research Ethics (REK) and registered in Clinicaltrials.gov. Ninety-three persons were included in the patient group and 92 in the control group. The patients were selected from two tertiary medical centers in central Norway in the period 2010 to 2015. General practitioners or specialists in otorhinolaryngology, pulmonary medicine, and internal medicine referred the patients to confirm their suspicions of sleep related disorders. They all underwent a sleep polygraph to verify the diagnosis. The controls were randomly chosen among hospital workers and workers outside of the hospital as part of their annual health check-up. All patients and controls signed a written consent before inclusion in the trial. Because of the skewness in gender distribution in OSA patients, we adjusted the gender in the control group to match the distribution in the patient group. The request to join the study as controls was done by registered nurses prior to clinical examination so they were blinded in regard to information on clinical examination of the nasal cavity. The inclusion criterion in the patient group was a verified diagnosis of OSA by a portable sleep polygraph test. We included patients and controls between the age of 18 and 75. In both groups the exclusion criteria were prior nasal surgery, use of decongestants or nasal steroids over the last 3 months, and evidence of chronic rhinosinusitis with or without nasal polyposis. Complaints of daytime sleepiness, excessive snoring, or observed respiratory distress by others were also considered exclusion criteria in the control group.

### 2.1. Sleep Polygraph Test

A portable sleep polygraph test (Embletta Diagnostic System, ResMed, San Diego, California, USA, and Nox Medical T3, ResMed, Reykjavík, Iceland) was performed on all patients to verify the OSA diagnosis. A drop in the peak signal by ≥90% of preevent baseline for ≥10 seconds using an oronasal sensor was the determining factor for apneas. Correspondingly, hypopnea was scored when the peak signal dropped by ≥30% of preevent baseline using nasal pressure for ≥10 seconds in association with ≥3% arterial oxygen desaturation. An apnea-hypopnea-index (AHI) > 5 per hour was considered abnormal. An experienced sleep physiologist examined all sleep reports manually prior to the diagnosis. The respiratory disturbance index and oxygen desaturation index were evaluated but did not form the basis for the OSA diagnosis in this study.

### 2.2. Sinonasal Outcome Test

Sinonasal Outcome Test-20 (SNOT-20) is a validated patient reported measure of health related QoL in sinonasal disease [4, 5]. The later modified version, SNOT-22, was still not validated in Norwegian at the onset of the trial. The patients were asked to grade 20 items on a scale from 0 (no complaints) to 5 (problem as severe as can be). The SNOT-20 score for each subject was defined as the mean value of the response to the 20 items. SNOT-20 is divided into four different subsets as described by Browne et al. [6]: rhinologic problems, ear and facial problems, sleep function, and psychological issues. These subdomains have been found to be methodologically sound and are believed to improve the precision of the questionnaire compared to reporting single SNOT-20 scores alone [5].

### 2.3. Visual Analog Scale

The patients and controls reported symptoms as nasal obstruction, headache, facial pain, facial pressure, reduced sense of smell, nasal discharge, sneezing, coughing, snoring, oral breathing, and reduced general condition on a 100 mm visual analog scale (VAS). 0 mm on the scale equals “no symptoms” and 100 mm represents “as troublesome symptoms as possible.” The use of VAS in assessment of nasal obstruction (NO-VAS) has been validated and there is a strong correlation between the subjective VAS for nasal obstruction and nasal resistance [7].

### 2.4. Peak Nasal Inspiratory Flow (PNIF)

PNIF is an established clinical tool for evaluating nasal function [8]. It has been validated as a simple and reliable procedure that corresponds strongly with reports of subjective nasal obstruction. A portable PNIF meter (in-check DIAL; Clement Clarke International, Harlow, Essex, UK) was used. The mean of three approved PNIF measurements was recorded before and after decongestion with topical xylometazoline (Otrivin® 1 mg/ml, Novartis, Basel, Switzerland) with the subjects in a sitting position and the head held in a level position. A mean value after three approved measurements of 120 L/min was considered normal. One control was unable to perform PNIF.

### 2.5. Statistical Analysis

All data in the tables are presented as mean, standard deviation (SD), and 95 percent confidence interval (95% CI). The mean values between the patient group and the control group were analysed using an independent samples *t*-test. We used linear regression analysis and one-way analysis of variance (ANOVA) with Bonferroni for multiple comparisons tests in the subgroup analysis and to evaluate the significance of demographic variables. In addition we used the Pearson correlation coefficient to evaluate the correlation between NO-VAS and PNIF. If we wanted to detect a difference in SNOT-20 of 0.2 between the patient group and the control group, with a power of 80% and a level of significance set at 0.05, we needed 100 patients in each group. The complete set of data was analysed using IBM SPSS version 23.0 (SPSS Inc., Chicago, Illinois, USA).

## 3. Results

The groups were matched in age, gender distribution, and educational level but there was a significant difference between the groups regarding weight and BMI as expected since weight is strongly associated with development of OSA [9]. However, BMI did not contribute in a significant way to the total SNOT score (*p* = 0.82) or VAS-NO score (*p* = 0.45) in the patient group. There was a relatively even distribution of heart disease and asthma/allergy in both groups (Table 1).

### 3.1. SNOT-20

The OSA patients had an impairment in sinonasal QoL compared to the control group, with mean SNOT-20 scores of 1.69 (SD 0.84) and 0.55 (SD 0.69), respectively, *p* < 0.001. Similarly, there were highly significant differences between the groups for all items except for ear pain (*p* = 0.11). The difference between the groups in the four subsets of SNOT-20 was also highly significant, with better outcomes in the control group (Table 2).

### 3.2. VAS

The total VAS score was 41,3 (SD 12,8) in the patient group and 15,6 (SD 13) in the control group (*p* < 0,001). In addition, the differences in the subsets of the VAS scores were highly significant with the exception of headache and pain (Table 3).

### 3.3. PNIF

**Figure 1:**
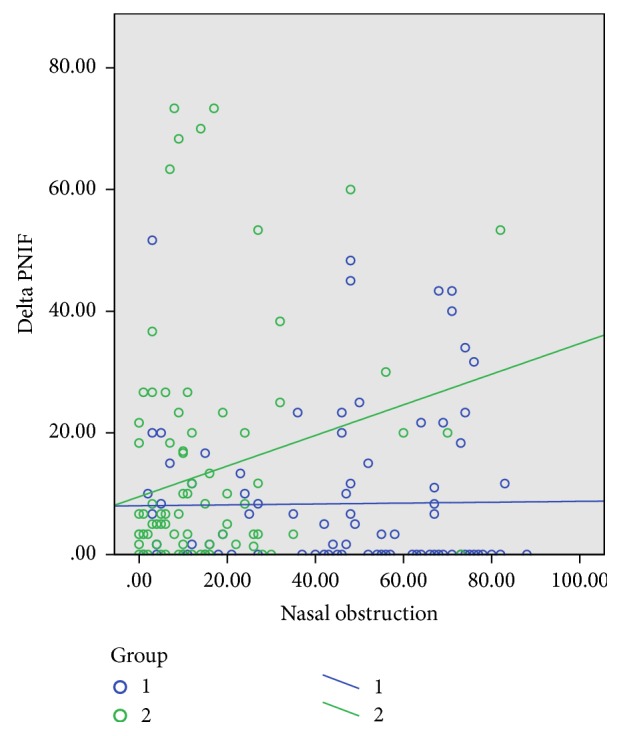
The change in PNIF before and after decongestion with xylometazoline compared to level of subjective nasal obstruction. Blue dots and line = OSA group. Green dots and line = controls. Delta PNIF = the absolute difference in PNIF at baseline and after decongestion (l/min). There is a significant positive correlation between VAS nasal obstruction and delta PNIF only in the control group.

There was a difference in PNIF scores between the OSA group and control group both at baseline (105 versus 117 l/min, *p* < 0,010) and after decongestion (113 versus 129 l/min, *p* < 0,010), respectively. There was a significant positive correlation between the absolute difference in PNIF before and after decongestion (delta PNIF) and NO-VAS scores in the control group (*p* = 0.026, *r* = 0.232) but not in the patient group (*p* = 0.891, *r* = 0.014) (Figure 1).

### 3.4. Subgroup Analysis

#### 3.4.1. AHI Severity

When stratifying the OSA group by AHI levels into mild (0–14,9), moderate (15–29,9), and severe (>30) we could see a positive correlation with total SNOT score, total VAS score, and NO-VAS score, although not statistically significant. When we looked at the four subdomains of SNOT-20, there was a significant difference only in the sleep subdomain (*F* (2,90) = 4.95, *p* < 0.01) between mild (mean 1.79), moderate (mean 2.69), and severe levels of AHI (mean 2.67). The multiple comparison test showed a significant difference between mild and moderate AHI (mean difference 0.89, *p* < 0.05, 95% CI [−1.64, −0.14]) and mild and severe AHI (mean difference 0.88, *p* < 0.05, 95% CI [0.14, 1.61]) but not between the moderate and severe levels of AHI (mean difference 0.02, *p* = 1.0, 95% CI [−0.55, 0.59]). When looking into the individual scores of the SNOT-20 questionnaire we could find a significant score in the subscore of “waking up at night” between mild (mean 1.88) and severe (mean 2.98) levels of AHI (mean difference 1.1, *p* < 0.05, 95% CI [−2.07, −0.13]). Regarding symptoms on VAS, there were significant differences in “snoring” between mild (mean 73.4) and severe (mean 88.4) levels of AHI (mean difference 15.0, *p* < 0.05, 95% CI [−27.1, −2.9]) and in “apnea” between both mild (mean 62.8) and severe (mean 86.3) levels of AHI (mean difference 23.5, *p* < 0.001, 95% CI [−37.3, −9.6]) and between moderate (mean 74.1) and severe (mean 86.3) levels of AHI (mean difference 12.2, *p* < 0.05, 95% CI [−22.9, −1.4]). Regarding the symptom of “headache” there was a significant level of difference only between moderate (mean 25.7) and severe (mean 40.5) levels of AHI (mean difference 14.7, *p* < 0.05, 95% CI [−29,4, −0.03]).

### 3.5. Age

We stratified the groups in age under 45, between 45 to 60, and over 60 but there were no differences between the age groups or between the patient group and controls regarding the subdomains of SNOT-20, total VAS score, or NO-VAS score.

### 3.6. Self-Reported Asthma/Allergy and Heart Disease

OSA patients with self-reported asthma/allergy had a significantly higher NO-VAS score (mean 57.2) compared to patients with self-reported heart disease (mean 21.7, mean difference 35.6, *p* < 0.01, 95% CI [11.7, 59.5]) but not compared to patients reporting no disease (mean 47.3, mean difference 9.9, *p* > 0.05, 95% CI [−5.9, 25.7]). In the “general health” symptom the asthma/allergy fraction in the OSA group scored significantly higher (mean 44.1) than the heart disease fraction (mean 16.2, mean difference 27.9, *p* < 0.05, 95% CI [4.47, 51.3]) and they also scored significantly higher compared to those who claimed not to have any disease (mean 27.9, mean difference 16.3, *p* < 0.05, 95% CI [0.80, 31.7]). In the control group there were no significant differences in VAS scores between the disease groups.

## 4. Discussion

We could demonstrate a marked reduction in sinonasal QoL in OSA patients compared to controls and an association between the degree of subjective nasal obstruction on VAS and the change in inspiratory flow in controls alone (Figure 1). The importance of normal nasal function in OSA patients has been noted in several publications in the past [10–15]. These studies generally tend to describe two important features regarding nasal patency and OSA. Firstly, they describe the facilitation of nasal continuous positive airway pressure devices or bilevel positive airway pressure devices (nCPAP/biPap) due to lower nasal resistance after medical and/or surgical treatment of nasal obstruction. Secondly, they describe the self-reported reduction in daytime sleepiness following a successful treatment of nasal obstruction in OSA patients. Despite the obvious effect of restoring nasal function on positive airway pressure treatment and subjective daytime sleepiness in patients, the effect on objective measures of obstructive sleep apnea keeps eluding us. These conflicting results raise more questions: Should we believe in QoL measures and postulate that the diagnostic tools we use today does not quite give a good enough measure of the daytime sleepiness associated with OSA? This view is supported by the increasing tendency to see obstructive sleep apnea as a result of a combination of not only the number of apneas and hypopneas, but also the nocturnal hypoxemia and respiratory disturbance index [16]. This has also led to the emerging notion of using OSA phenotyping to decide on specific treatment options [17]. The other question will be to see whether sinonasal characteristics differ not only in regard to OSA severity but also when compared to a supposedly healthy cohort.

In our study we could observe that, within the OSA group, the total SNOT scores and VAS scores were positively correlated to the severity of AHI. Although the differences did not reach the chosen level of significance it indicates a clear association between nasal complaints and severity of disease. This verifies the results in the study by Kuan et al. where sinonasal complaints evaluated by the SNOT-22 score seemed to be correlated to OSA severity [1].

When we expand our view and compare the OSA group to a healthy cohort, we find significant differences between groups for all symptoms given on VAS and all items in SNOT-20 except ear pain. All the four SNOT-20 subdomains showed a highly statistical difference between the OSA group and the controls, and the subanalysis showed a positive correlation in the subdomain of sleep with severity of AHI. This is consistent with earlier studies showing the association between cognitive impairment and OSA severity [18]. The level of difference in both SNOT-20 and VAS is stronger between the patient group and the controls than between the different levels of AHI severity in the patient group. We believe that this points to a strong association between obstructive sleep apnea and nasal obstruction regardless of severity measured by AHI. This does not, however, yield any information as to whether it is a causative association or merely a concurrent phenomenon, but it falls in line with earlier studies that demonstrate that lower nasal cavity volumes and impairment of nasal function are associated with development of OSA [19]. Treatment of septal deviations in OSA patients has also been shown to lead to better QoL and relief of nasal symptoms compared to healthy individuals which gives more strength to this observed association [20]. The differences in total SNOT score and total VAS score were more pronounced between patients with a mild and moderate AHI level than between patients with a moderate and severe AHI level. This might suggest that nasal involvement has a greater impact on milder forms of OSA and that expectations of possible curative treatments of nasal obstruction in OSA should be limited to this group.

Self-reported asthma and allergy in the OSA group seemed to be correlated to higher VAS-NO scores compared to patients with heart disease and are coherent with studies indicating a synergistic effect between asthma and OSA [21] in much the same way as seen with chronic obstructive pulmonary disease [22]. This synergistic effect is also reflected in the subgroup analysis where there were significant differences in the VAS symptom “general health” in OSA patients with asthma/allergy compared to nonallergy/nonasthmatic OSA patients. In the control group a higher VAS nasal obstruction score was positively correlated to the absolute difference in PNIF before and after decongestion, reflecting their ability to increase nasal function as the nasal mucosal swelling was reduced. The higher the NO-VAS score, the higher the change in PNIF after decongestion. This correlation was not seen in the OSA group (Figure 1). The inability to increase PNIF in the patient group after decongestion, as well as the reported higher nasal obstruction scores in asthma and allergic patients, can be supportive of the idea of an inflammatory component in the nasal mucosa that is not affected by decongestion by xylometazoline or that there is a higher bone to mucosa ratio in the nasal valve area of the nose in OSA patients. Reports on proinflammatory cytokines like interleukin-6 (IL 6) are also suggestive of an association between OSA with objective excessive daytime sleepiness and low grade inflammation [23]. Asthmatics are known to have a reduced PNIF compared to nonasthmatics [24] and asthma in OSA patients might be considered a mediator in the reduction of PNIF in OSA patients.

### 4.1. Strengths and Limitations

The major strength of this study is the pragmatic study design based on prospective data in an everyday clinical setting and the relatively large study population. A limitation of SNOT-20 compared to SNOT-22 is that the latest version of the questionnaire has two additional questions on nasal congestion and decreased sense of smell/taste. Even though our study showed a marked difference in all twenty subsets, information on differences in problems with olfactory function and nasal blockage would have given additional value to the study. Our control group was recruited at random from occupational check-ups and from coworkers at the hospital. Although they made the inclusion criteria, they did not undergo a sleep polygraph to exclude the OSA diagnosis. But the elimination of a potential OSA fraction among controls would only give strength to the differences between the groups rather than weaken them. The self-reported asthma/allergy and heart disease might be prone to misclassification bias and the results may be underestimated.

## 5. Conclusion

Sinonasal QoL is significantly reduced in OSA patients compared to a normal cohort measured by SNOT-20, subdomains of SNOT-20, and nasal obstruction VAS score. The subanalysis showed a positive, but not statistically significant, correlation between AHI levels and QoL measures. Subanalysis also showed that the ability to increase nasal inspiratory flow in OSA patients was unaffected by xylometazoline compared to controls, suggesting that additional factors other than AHI sublevels might increase sinonasal complaints in OSA patients. A possible mechanism could be that OSA patients have a smaller inlet area of the nose caused by nasal inflammatory pathways or a reduction of the skeletal framework that constitutes the nasal valve area. Due to its large impact on QoL, relief of nasal obstruction should be a concern in treatment of OSA patients.

## Figures and Tables

**Table 1:** Patient demographics.

	OSA (*N* = 93)	Controls (*N* = 92)	*p* value
Gender			
Female (%)	25 (26,9)	23 (25,0)	0.77
Male (%)	68 (73,1)	69 (75)	
Mean age, years (range)	49.3 (27–72)	46.0 (20–69)	0.06
Mean height, m (SD)	1.77 (0.10)	1.78 (.09)	0.40
Mean weight, kg (SD)	95.4 (16.7)	82.4 (14.6)	<0.01
Education, years (%)			
<9	12 (12.9)	12 (13.0)	0.72
10–12	28 (30.1)	24 (26.1)	
>13	53 (57.0)	56 (60.9)	
Disease, *n* (%)			
Heart disease	9 (9.7)	8 (8.7)	0.80
Allergy	17 (18.3)	10 (10.9)	0.15
Mean BMI, kg/m^2^ (SD)	30.3 (4.3)	25.8 (3.5)	<0.01

**Table 2:** Scores for the Sinonasal Outcome Test (SNOT-20) in the OSA group and controls. Data presented as mean (SD) and 95% CI.

Question	OSA group (*n* = 93)	Control group (*n* = 92)	*p* value	95% CI
Need to blow nose^a^	1.58 (1.32)	0.63 (0.91)	<0.001	(0.6, 1.3)
Sneezing^a^	1.39 (1.26)	0.61 (0.81)	<0.001	(0.5, 1.1)
Runny nose^a^	1.20 (1.19)	0.40 (0.68)	<0.001	(0.5, 1.1)
Cough	1.41 (1.36)	0.40 (0.76)	<0.001	(0.7, 1.3)
Postnasal discharge^a^	1.00 (1.34)	0.23 (0.58)	<0.001	(0.5, 1.1)
Thick nasal discharge^a^	1.20 (1.35)	0.42 (0.83)	<0.001	(0.5, 1.1)
Ear fullness^b^	1.26 (1.29)	0.58 (1.06)	<0.001	(0.3, 1.0)
Dizziness^b^	0.96 (1.38)	0.46 (0.93)	0.004	(0.2, 0.8)
Ear pain^b^	0.51 (0.95)	0.29 (0.82)	0.106	(−0.1, 0.5)
Facial pain/pressure^b^	0.63 (1.12)	0.25 (0.72)	0.006	(0.1, 0.7)
Difficulty falling to sleep^c^	1.32 (1.55)	0.49 (1.05)	<0.001	(0.5, 1.2)
Wake up at night^c^	2.72 (1.39)	0.91 (1.35)	<0.001	(1.4, 2.2)
Lack of good night's sleep^c^	3.53 (1.26)	0.99 (1.51)	<0.001	(2.1, 2.9)
Wake up tired	3.32 (1.24)	1.46 (2.00)	<0.001	(1.4, 2.4)
Fatigue^d^	2.44 (1.56)	0.60 (1.15)	<0.001	(1.5, 2.2)
Reduced productivity^d^	2.44 (1.56)	0.61 (1.15)	<0.001	(1.4, 2.2)
Reduced concentration^d^	2.52 (1.54)	0.69 (1.16)	<0.001	(1.4, 2.2)
Frustrated/restless/irritable^d^	2.12 (1.54)	0.59 (1.10)	<0.001	(1.1, 1.9)
Sad^d^	1.16 (1.33)	0.27 (0.61)	<0.001	(0.6, 1.2)
Embarrassed^d^	0.65 (1.15)	0.10 (0.39)	<0.001	(0.3, 0.8)
Subset				
Rhinologic^a^	1.28 (0.96)	0.46 (0.58)	<0.001	(0.6, 1.1)
Ear/facial^b^	0.83 (0.88)	0.39 (0.73)	<0.001	(0.2, 0.7)
Sleep function^c^	2.52 (1.07)	0.79 (1.18)	<0.001	(1.4, 2.1)
Psychological function^d^	1.88 (1.19)	0.49 (0.83)	<0.001	(1.1, 1.7)
Mean SNOT-20	1.69 (0.84)	0.55 (0.69)	<0.001	(0.9, 1.4)

^a^Questions = rhinologic subset, ^b^Questions = ear/facial subset, ^c^Questions = sleep functions subset, and ^d^Questions = psychological subset.

**Table 3:** Visual Analog Scale (VAS) scores for sinonasal symptoms in OSA patients and controls. Data presented as mean (SD) and 95% CI.

Symptoms	OSA group (*n* = 93)	Control group (*n* = 92)	*p* value	95% CI
Nasal blockage	46.2 (25,5)	14.1 (17.1)	<0.001	(25.7, 38.3)
Oral breathing	55.5 (26.2)	19.0 (23.1)	<0.001	(29.4, 43.7)
Snoring	84.2 (17.5)	36.1 (31.7)	<0.001	(40.6, 55.5)
Sleep apnea	77.5 (21.0)	14.2 (22.0)	<0.001	(57.1, 69.6)
Nasal discharge	28.8 (24.0)	12.9 (17.0)	<0.001	(9.9, 22.0)
Headache	32.5 (27.1)	20.8 (24.2)	0.002	(4.3, 19.2)
Midfacial pain	16.0 (20.5)	9.8 (15.9)	0.024	(0.8, 11.5)
Rhinosinusitis	16.6 (20.1)	4.8 (8.0)	<0.001	(7.3, 16.2)
Coughing	32.1 (25.3)	11.6 (14.0)	<0.001	(14.6, 26.4)
Sneezing	43.8 (59.0)	19.4 (19.8)	<0.001	(11.6, 37.2)
Reduced general health	29.5 (24.3)	11.6 (19.6)	<0.001	(11.5, 24.3)
Total VAS score	41.3 (12.8)	14.7 (14.4)	<0.001	(22.7, 30.6)
